# Contention-Aware Adaptive Data Rate for Throughput Optimization in LoRaWAN

**DOI:** 10.3390/s18061716

**Published:** 2018-05-25

**Authors:** Sungryul Kim, Younghwan Yoo

**Affiliations:** School of Electrical and Computer Engineering, Pusan National University, Busan 46241, Korea; xmfhxm12@pusan.ac.kr

**Keywords:** adaptive data rate, contention-aware, gradient projection method, LoRaWAN, throughput optimization

## Abstract

In Long Range Wide Area Network (LoRaWAN), the data rate of the devices can be adjusted to optimize the throughput by changing the spreading factor. However, the adaptive data rate has to be carefully utilized because the collision probability, which directly affects the throughput, is changed according to the use of spreading factors. Namely, the greater the number of devices using the same spreading factor, the greater the probability of collision, resulting in a decrease of total throughput. Nevertheless, in the current system, the only criteria to determine the data rate to be adjusted is a link quality. Therefore, contention-aware adaptive data rate should be designed for the throughput optimization. Here, the number of devices which can use a specific data rate is restricted, and accordingly the optimization problem can be regarded as constrained optimization. To find an optimal solution, we adopt the gradient projection method. By adjusting the data rate based on the retrieved set of optimal data rate, the system performance can be significantly improved. The numerical results demonstrate that the proposed method outperforms the comparisons regardless of the number of devices and is close to the theoretical upper bound of throughput.

## 1. Introduction

During the past decade we became witnesses of the evolution of the Internet of Things (IoT) technology. The IoT provides a useful service platform by connecting various things such as devices, objects, animals and plants [1]. Recently, the value of small data has been garnering attention in the IoT industry [2,3]. The things that transmit a small amount of data such as temperature, humidity, weight, and location are classified as small things, and the network composed of such small things is called the Internet of Small Things (IoST). Since the exchanged data size in IoST is very small, a low-cost and low-performance processor is able to sufficiently meet the application requirements. In addition, the small things sparsely transmit the sensing data, and consequently the device can operate for several years with just one battery. These advantages encourage developers and companies to freely release their IoT-based services.

The IoST network can be divided into two categories, local area network and wide area network. Local area network, which has a short communication range, provides a relatively high data rate, e.g., Radio Frequency Identification (RFID) system, Bluetooth Low Energy (BLE) beacon, and Near Field Communication (NFC). Meanwhile, Low-Power Wide Area Network (LPWAN) is characterized by the long-range communication and the low data rate. The gateway is able to use the sensing data from the end-devices distributed in a city, thus the LPWAN technology is suitable for the purpose of collecting small amounts of data scattered over a large area. For instance, metering such as electricity and gas, remote management of livestock, smart traffic management, and waste collection scheduling. Moreover, this can be adopted for the networking of Unmanned Aerial Vehicle (UAV)-based system, and those collaborative operations will create innovations [4,5]. Accordingly, many standards related to the LPWAN have been introduced, and LoRaWAN and SIGFOX are considered as the leading group [6,7].

The LoRaWAN provides the higher data rate as compared with SIGFOX while consuming the lower amount of energy. Furthermore, in contrast to the SIGFOX adopting Gaussian Frequency-Shift Keying (GFSK) and Binary Phase Shift Keying (BPSK) for the modulation [8], the LoRaWAN adopts Chirp Spread Spectrum (CSS), and it makes LoRa signal more robust to the interference and Doppler shifting [9]. Meanwhile, in the LoRaWAN, the signal length is determined by Spreading Factor (SF) indicating the number of encoded bits per symbol. In detail, the bigger SF is, the longer the signal length becomes. Since the minimum Signal-to-Noise Ratio (SNR) required for successful decoding increases as the signal length increases, the devices should use the larger SF as it increases communication range. One notable thing is that the data rate is also determined by the SF. In other words, the gateway can control the data rate of each device by adjusting SF, i.e., Adaptive Data Rate (ADR) is permitted [6]. The current LoRaWAN standard gives a simple guideline; the gateway is able to change the data rate according to the link quality retrieved from SNR. However, the detailed descriptions for the ADR have not yet been concretely established.

This paper proposes a contention-aware adaptive data rate to maximize the total throughput in the LoRaWAN. For higher throughput, it seems to be reasonable that all end-devices just use the smallest SF among available SFs. However, the more end-devices that use the same SF, the greater the probability of collision, resulting in decreasing throughput [10]. This is due to the fact that the signals generated by different SFs can be separated even though they overlap at the gateway owing to its orthogonality. In contrast, when the signals generated by the same SF are overlapped, the collision happens. Therefore, not only link quality, but also the impact of changed SFs on the contention has to be considered in ADR. For this purpose, we define the total throughput as an objective function with respect to the number of devices using specific SFs. Accordingly, finding a set of the number of devices per SF that maximizes the objective function is the goal of this paper. That is, we derive the theoretical optimal throughput that can be achieved by adjusting only the data rate of the devices. Here, the available SF of each device is limited, depending on the communication range. Therefore, the maximization problem is categorized into the constrained optimization. To solve the problem, we adopt the gradient projection method that finds the optimal solution while keeping the constraints. As a result, owing to the mitigation of the contention, our method can achieve optimal throughput in the given environments. For the performance evaluation, we assume two comparable ADR schemes based on the guidance of the LoRaWAN. In the first scheme, all devices change their data rate to the fastest data rate by using the smallest possible SF. This called the naive approach. This can be considered to be very similar to the method described in the current standard. The second approach is that the number of devices using a specific SF is made equal, named as the uniform approach. This can alleviate the contention problem by inducing the devices to use the SFs evenly. The numerical results show that our method outperforms the comparisons.

The contribution of this paper is twofold as follows.

- To the best of our knowledge, this is the first ADR considering the contention problem in the LoRaWAN.
- For the optimization, we adopt the gradient projection method. This allows the gateway to easily find the optimal set under the constraints regardless of the number of end-devices deployed in the network.

The rest of this paper is organized as follows. Section 2 briefly introduces basic knowledge of the LoRaWAN. Section 3 describes the contention problem caused by the adjustment of data rate. Section 4 presents the proposed method, contention-aware ADR and how to find the optimal solution with the gradient projection method. The numerical results show that the proposed method supports higher throughput as compared with the comparisons and it is close to the upper-bound of the performance. Finally, we conclude our paper with a summary and future works.

## 2. Background Knowledge

This section discusses overall features of the LoRaWAN. First, we describe the modulation in the LoRaWAN, CSS, and illustrate how to adaptively adjust the data rate by changing the SF. In addition, pure ALOHA protocol and Frequency Hopping Spread Spectrum (FHSS) are briefly explained to understand how the LoRaWAN handles the multiple access. Meanwhile, the LoRaWAN standards are slightly different from region to region, and we consider EU863-870 (European standard) which operates in the Industrial Scientific Medical (ISM) band, 863–870 MHz.

### 2.1. Chirp Spread Spectrum

In the LoRaWAN, CSS modulation is used to generate the signal. The frequency of the CSS signal either linearly increases or decreases over the whole bandwidth, and this makes the signal robust against the interference and the Doppler shifting [11,12]. The number of bits encoded per symbol is decided by the spreading factor ranging from 7 to 12 in CSS modulation. Let $R_{s}$ and $R_{b}$ denote the symbol rate and bit rate respectively, and then the SF can be defined as below:

(1) $$SF=\frac{R_{b}}{R_{s}}.$$

In addition, the symbol length $T_{s}$ is given as

(2) $$T_{s}=\frac{2^{SF}}{BW},$$

where BW indicates the system bandwidth. The symbol rate is inverse of the symbol length, consequently (1) can be modified as follows.

(3) $$R_{b}\left(SF\right)=SF\times R_{s}=\frac{SF}{2^{SF}}BW.$$

The data rates and symbol lengths according to the used SF are presented in Table 1.

**Figure 1:**
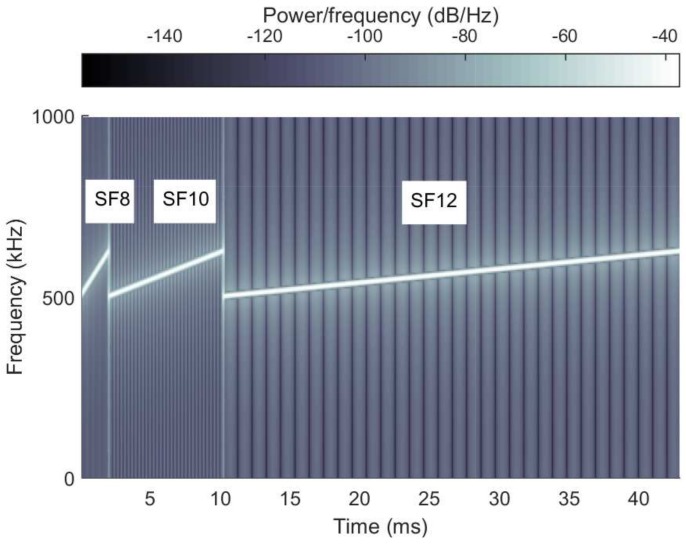
The spectrogram of the symbols generated by SF8, SF10 and SF12.

Figure 1 shows the spectrogram of the symbol in the case of SF8, SF10, and SF12 respectively. As shown in the figure, the symbol length, i.e., Time on Air (ToA) is proportional to SF. Typically, as the symbol duration increases, the required SNR for decoding decreases. Therefore, the device near the gateway is able to use all kinds of SF owing to the loose constraint of SNR, but the device far from the gateway is forced to use only large SFs. If the gateway estimates that a certain device is able to use the smaller SF as compared with the current one, it tries to adjust the data rate to maximize the total throughput, and this is referred to as ADR in the LoRa system.

### 2.2. Adaptive Data Rate

The LoRaWAN allows the devices to use any of the possible data rates. By adjusting the data rate of each device, the throughput of the LoRaWAN can be optimized. As mentioned above, the data rate is determined by the used SF, consequently adjusting the data rate to select one of SFs to be used among available SFs.

ADR is initiated by the gateway. The gateway retrieves available minimum SF based on the SNR. After that, the gateway informs the device of the target SF via a MAC command named LinkADRReq. One notable thing is that this mechanism is applicable only to the devices that set ADR bit to 1, otherwise the ADR cannot be applied regardless of the link quality [6].

Meanwhile, after adjusting the data rate, it has to be verified whether the changed SF is appropriate because it is possible that the gateway may excessively recommend too small of SF for the data rate enhancement. To verify that the gateway still successfully receives the transmitted signal generated by the adjusted SF, the device increments a counter, referred to as ADR_ACK_CNT by one for each transmission, and this counter is reset whenever the device receives the response from the gateway. If ADR_ACK_CNT reaches the pre-defined threshold, ADR_ACK_LIMIT, due to the consecutive communication failures, the device sets ADRACKReq on the MAC command field in the next transmission. If there is no response from the gateway within a time duration specified at ADR_ACK_DELAY, the changed SF is considered as too small, and thus the devices increment SF for more reliable communication. This procedure is repeated until the device finds an appropriate SF or the adjusted SF is equal to the default one.

In EU863-870, the default setting of ADR_ACK_LIMIT is 64. This means that once the data rate is incorrectly adjusted, it takes a long time to be corrected at the proper data rate. Therefore, the data rate should be carefully adjusted from the perspective of the entire network rather than from the perspective of the individual devices.

### 2.3. Medium Access Control in the LoRaWAN

The strategies to control the multiple medium access can be explained with pure ALOHA, FHSS, and signal orthogonality. Due to the philosophy of the LoRaWAN, i.e., low performance and low power, the complicate MAC protocols are not preferred [5]. Instead of some advanced MAC protocols, the LoRaWAN adopts Pure ALOHA, i.e., broadcasting and reply mechanism [13]. The slotted ALOHA is also inappropriate because it requires time synchronization consuming additional battery and computational resources. For the more efficient medium access, the Listen Before Talk (LBT) mechanism is applicable, but it is ignored in this paper to focus on the impact of ADR on the throughput optimization.

Along with the pure ALOHA, the LoRaWAN uses FHSS to mitigate the collision problem [14]. Therefore, each device attempts to send their packet on a channel randomly selected every transmission. Even though the multiple signals arrive at the gateway at the same time, they can be successfully decoded as long as the used channels are different each other. According to EU863-870, a total of six channels are available. Three channels working on 868.10, 868.30, 868.50 MHz are regarded as default channels and all devices are obliged to support those channels. In addition, the gateway should always be listening on the channels. The remaining channels working on 868.10, 868.30, 868.50 MHz are mainly used by the devices to broadcast the JoinReq message.

One notable point is that chirp rate describing how fast the frequency changes during the symbol duration can also be utilized to avoid packet collision. The signal generated with different chirp rates are near orthogonal each other, thus they can be separated, even though the arrival times and the used channels are the same [15]. The chirp rate is defined as

(4) $$k=\frac{BW^{2}}{2^{SF}}.$$

As shown in the above equation, for the different chirp rates, we can change the bandwidth or SF. Actually, the bandwidth of all channels is fixed at 125 kHz, consequently the only method to get a unique chirp rate is to adjust SF, which determines the data rate.

**Figure 2:**
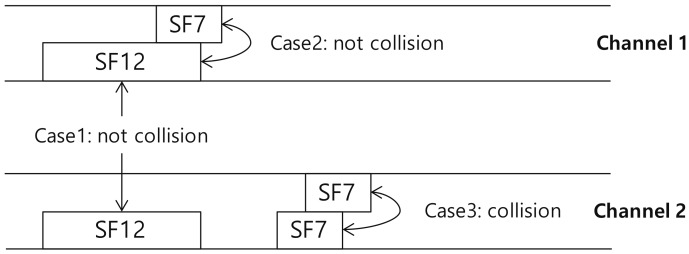
Multiple access control in the pure-ALOHA based the LoRaWAN.

Figure 2 illustrates multiple access control in the LoRaWAN. For simplicity, we assume that the number of available SFs is two, SF7 and SF12. In case 1, although two signals generated with SF12 arrive at the same time, they can be successfully decoded because of different channel usage. In case 2, owing to the orthogonality, the collision can be avoided, even though multiple devices use the same channel. However, the collision occurs in case 3 since the two signals generated with the same SF (SF7) arrive on the same channel. In conclusion, a collision occurs only when the multiple signals generated with the same SF overlap on the same channel.

## 3. Impact of ADR on the Contention

As mentioned above, the SF is used not only to adjust the data rate, but also for the multiple access control. Even if the devices are allowed to use the higher data rates, it is useless if excessive collisions occur. Therefore, we have to investigate the impact of ADR on the collision probability and corresponding throughput.

As long as the used SF or channel are different, collision does not occur, consequently the LoRaWAN can be regarded as a set of $N_{c}\times N_{s}$ pure ALOHA-based sub-networks which independently operate each other, where $N_{c}$ and $N_{s}$ is the number of available channels and SFs respectively. Let *N* denote the total number of devices, and $n_{i}$ denote the number of devices using SF *i*. We assume that the devices select a channel with the same probability, and then the number of devices which attempt to transmit a packet using SF *i* and channel *j* can be approximated to

(5) $$n_{i,j}\approx \frac{n_{i}}{N_{c}}.$$

ToA for sending a packet of length *L* can be calculated as below:

(6) $$t_{i}=\frac{L}{R_{b}\left(i\right)}.$$

Under the assumption that the transmission probability of all devices is equal to $P_{tx}$, the average number of packets arriving on channel *j* within the time interval $t_{i}$ is denoted by $\lambda_{i,j}$, and it is calculated by

(7) $$\lambda_{i,j}=P_{tx}n_{i,j}.$$

Assuming that the distribution of packet arrival follows the Poisson process, the probability that *k* packets arrive within $t_{i}$ can be represented by

(8) $$P\left(k;\lambda_{i,j}\right)=\frac{{\left(t_{i}\lambda_{i,j}\right)}^{k}}{k!}e^{-t_{i}\lambda_{i,j}}.$$

For the successful transmission, only one device attempts to send a packet when the channel is idle state, and the others must keep silent during the vulnerable period, i.e., 2$t_{i}$. Therefore, in ALOHA-based communication, the success probability $P_{s}$ can be written as

(9) $$P_{s}\left(i,j\right)=e^{-2t_{i}\lambda_{i,j}}=e^{-2G(i,j)}.$$

where G(i,j), which is calculated by $t_{i}\lambda_{i,j}$, is the traffic load on the sub-network defined by SF *i* and channel *j*. Based on (9), we can induce the throughput of a sub-network defined by the SF *i* as below:

(10) $$S_{i}=\sum_{j=1}^{N_{c}}G\left(i,j\right)P_{s}\left(i,j\right).$$

Finally, the total throughput can be calculated roughly as the superposition of independent sub-networks, that is,

(11) $$S=\sum_{i=1}^{N_{s}}S_{i}.$$

**Figure 3:**
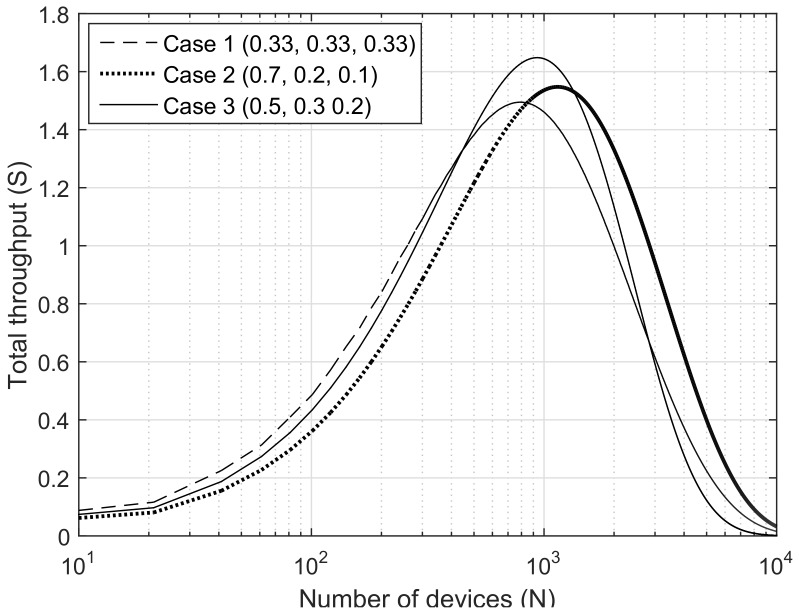
The comparison of the throughput according to the SF distribution.

To give an insight into the relationship between the number of devices using the same SF and the throughput, we define the ratio of devices using SF *i* as $\alpha_{i}$(calculated by $\frac{n_{i}}{N}$), and then the distribution of used SFs can be presented by a combination of $\alpha_{i}$. Assuming that SF7, SF8 and SF9 are available, we examine three cases of ($\alpha_{7}$, $\alpha_{8}$, $\alpha_{9}$); Case1: (0.33, 0.33, 0.33), Case2: (0.7, 0.2, 0.1) and Case3: (0.5, 0.3, 0.2) for ($\alpha_{7}$, $\alpha_{8}$, $\alpha_{9}$). Case1 considers that all SFs are used uniformly. In Case2, a majority device might use the highest data rate by using SF7. Meanwhile, in Case3, most traffic load is adequately distributed to the spreading factor domains. From the results shown in Figure 3, we can notice two important facts. First, it is not always effective for a majority device to simply use the highest data rate by applying SF7. The throughput presented in case 2 is less than the others until the number of devices is over 1000. This implies that although the devices are able to enhance their data rate using the small SF, this may need to be refrained from in some cases. Second, there is no sure way of providing the highest throughput regardless of the number of devices. Case1 is the best policy when the number of devices is small, and Case3 outperforms others when the number of devices is about 1000, and finally Case2 is the best when the number of devices is large. Therefore, the data rate adaptation has to be dynamically applied depending on the given network state.

## 4. Contention-Aware Adaptive Data Rate

The maximization of the throughput can be translated into an optimization problem. In particular, the smallest available SF of each device is restricted according to the communication distance, consequently this problem can be categorized into constrained optimization. In this section, we define the throughput as an objective function with respect to the number of devices using SF *i*, and present how to find the optimal solution maximizing the total throughput based on the gradient projection method.

### 4.1. Objective Function

Let $X=\left\{n_{7},n_{8},\ldots ,n_{12}\right\}$ denote a set of the number of devices using SF *i*, the total throughput presented in (11) can be modified as a function f(X) using (9), that is,

(12) $$\begin{array}{cc} S=f(X) & =\sum_{j=1}^{N_{c}}t_{7}P_{tx}n_{7,j}e^{-2t_{7}P_{tx}n_{7,j}}+\ldots +t_{12}P_{tx}n_{12,j}e^{-2t_{12}P_{tx}n_{12,j}}\\ & \approx N_{c}\left(t_{7}P_{tx}n_{7}e^{-2t_{7}P_{tx}n_{7}}+\ldots +t_{12}P_{tx}n_{12}e^{-2t_{12}P_{tx}n_{12}}\right). \end{array}$$

The f(X) is the objective function, and finding a matrix X that maximizes the function output is the goal of our study. This can be presented as below:

(13) $$\underset{X\in \chi }{maximize}\;f\left(X\right)$$

where χ is all feasible sets of $\alpha_{i}$.

The searching spaces are bounded by several constraints. Most of all, the sum of all $n_{i}$ should be equal to the total number of devices, i.e., $\sum_{i=1}^{N_{s}}n_{i}=N$. In addition, $n_{i}$ is bounded because some devices cannot use any kind of SF. For instance, a device near the gateway is allowed to SF7. However, a device deployed at the edge is forced to use just large SFs such as SF11 or SF12. To understand the constraints in the optimization, we suppose that a total of four devices are deployed in the LoRaWAN. Device 1 and 2 are located close to the gateway, device 3 is at the middle, and device 4 is at the edge, and the smallest available SF in each device is presented in Table 2. This table might be maintained in the gateway. By counting the devices with the available smallest SF *i*, we can obtain the upper bounds as shown in Table 2. This means that the number of devices using the SF7 cannot be in excess of 2. In the same manner, the number of devices using the SF8 cannot excess 3. Over those constraints, the optimal solution should be found. Let $u_{i}$ denote the upper bound of $n_{i}$, and then all the constraints in the optimization can be represented by

(14) $$\begin{array}{cc} g_{1}\left(X\right) & =n_{7}+n_{8}+n_{9}+n_{10}+n_{11}+n_{12}=N\\ g_{i}\left(X\right) & =\sum_{j=7}^{i+5}n_{j}\leq u_{i},\;2\leq i\leq 6 \end{array}$$

Here, $g_{1}$ is called equality constraint and the others (from $g_{2}$ to $g_{6}$) is called inequality constraint. As a result, the total throughput can be maximized by deriving X that maximizes the objective function while satisfying the constraints expressed in (14).

### 4.2. Gradient Projection Method

**Figure 4:**
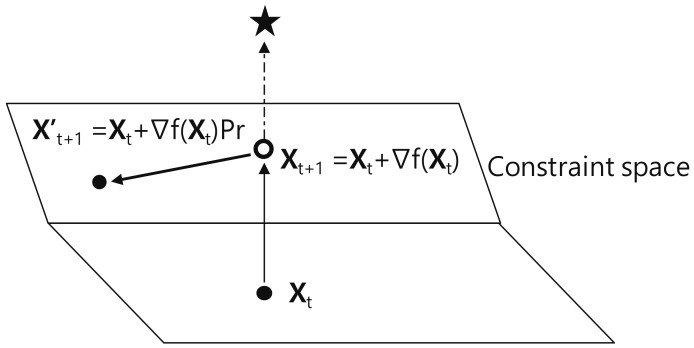
The comparison of the throughput according to the SF distribution.

This paper adopts the gradient projection method to solve the constrained optimization problem [16]. This algorithm is improved from the gradient descent method that finds the optimal value by iteratively updating the current solution in a small gradient direction. Both algorithms are based on the fact that the optimal is found at the point where the gradient of the objective function is zero. In particular, in gradient projection method, the next solution is always examined to determine if it violates the given constraints. If it is determined that the constraint will be violated, the next solution is projected into the constraint space. Figure 4 geometrically illustrates the process finding an optimal in the gradient projection method. The solution $X_{t}$ found at the *t*th time step has to be updated in the direction in which the optimal marked as star exists. However, $X_{t+1}$ is out of the constraint spaces, i.e., constraint violation occurs. To find the optimal on the feasible space, $X_{t+1}$ is projected into the constraint space by using the projection matrix Pr. In conclusion, $X_{t}$ is updated to the next projected solution, $X_{t+1}^{\prime }$.

To determine the direction of updating, partial derivatives of the objective function with respect to $n_{i}$ should be derived as follows:

(15) $$\frac{\partial f(X)}{\partial n_{i}}=N_{c}\left(t_{i}P_{tx}e^{-2t_{i}P_{tx}n_{i}}-2t_{i}^{2}P_{tx}^{2}n_{i}e^{-2t_{i}P_{tx}n_{i}}\right).$$

The partial derivative can be expressed in form of a matrix, that is,

(16) $$D\left(X\right)=\left[\begin{array}{c} \frac{\partial f(X)}{n_{7}}\\ \;\ldots \\ \frac{\partial f(X)}{n_{12}} \end{array}\right].$$

In the gradient descent method, the gradient derived from the partial derivative is directly applied to update the current solution. However, in the gradient projection method, it is validated that the updated solution still satisfies all constraints. For this purpose, the constraint equations expressed in (14) is modified to matrix form as follows:

(17) $$\left[\begin{array}{c} g_{1}\left(X\right)\\ g_{2}\left(X\right)\\ g_{3}\left(X\right)\\ g_{4}\left(X\right)\\ g_{5}\left(X\right)\\ g_{6}\left(X\right) \end{array}\right]=\left[\begin{array}{cccccc} 1 & 1 & 1 & 1 & 1 & 1\\ 1 & 0 & 0 & 0 & 0 & 0\\ 1 & 1 & 0 & 0 & 0 & 0\\ 1 & 1 & 1 & 0 & 0 & 0\\ 1 & 1 & 1 & 1 & 0 & 0\\ 1 & 1 & 1 & 1 & 1 & 0 \end{array}\right]\left[\begin{array}{c} n_{7}\\ n_{8}\\ n_{9}\\ n_{10}\\ n_{11}\\ n_{12} \end{array}\right]=\left[\begin{array}{c} N\\ u_{7}\\ u_{8}\\ u_{9}\\ u_{10}\\ u_{11} \end{array}\right].$$

For simplicity, it can be rewritten as

(18) $$G\left(X\right)=AX^{T}-U.$$

The constraint types can be divided into tight constraint and loose constraint, and they can be distinguished by observing G(X). If the *k*th component of G(X) is zero or a positive value, it is regarded as a tight constraint. It should be noted that the equality constraint is always treated as a tight constraint. In addition, except for the tight constraints, the remainder is considered a loose constraint. For the projection, the projection matrix is defined as

(19) $$\mathrm{Pr}=I-A_{T}^{\prime }{\left[A_{T}A_{T}^{\prime }\right]}^{-1}A_{T}$$

where I is the identity matrix and $A_{T}$ is the tight constraint matrix containing all row elements of A that are found to be tight constraint. If the tight constraints are founded, the corresponding gradients have to be projected into constraint space, namely,

(20) $$D^{\prime }\left(X\right)=D\left(X\right)\mathrm{Pr},$$

After projecting the gradients, the forwarding step size should be decided. It is similar to the step size used in the gradient descent method. The step size should also be bounded by the constraints, and it can be induced by calculating a matrix H as follows:

(21) $$H=-G\left(X\right)./A_{L}$$

where the notation ./ means element-wise division and $A_{L}$ is a matrix containing the loose constraints. Between the smallest positive element in H and 1, the smaller value is selected for the step size λ to be used in the update; it is expressed by

(22) $$\lambda =min(H_{min}^{+},1).$$

It is noted that in the case of minimization problem, λ is determined by the smallest negative in H. Finally, the current solution $X_{t}$ is updated as follows:

(23) $$X_{t+1}=X_{t}+\lambda D^{\prime }\left(X\right).$$

The updating rule shown in (23) is continuously repeated until it meets the termination condition. Typically, the algorithm stops when the norm of the gradients is less than the pre-defined threshold $\theta_{d}$. The termination condition can be presented by

(24) $$∥D(X)∥\leq \theta_{d}.$$

When the current solution satisfies (24), the algorithm stops and the solution found at that time is regarded as the optimal solution. This solution indicates how many devices must use a particular SF for the optimal throughput. The gateway adjusts the data rate of each device based on the retrieved solution. The pseudo code of the algorithm is shown in Algorithm 1.
**Algorithm 1** Gradient Projection Method to find an optimal distribution of *SF*s.**Require:** 1:*N*: Total number of devices 2:U: A matrix containing the upper bound of the number of devices can be used SFi 3:$\theta_{d}$: Threshold for norm of gradients 4:A: Constraint matrix 5:f(X): Objective function 6: 7:**procedure**Initialization 8:  $D\left(X\right)\leftarrow f{\left(X\right)}^{\prime }$,                        ▷ get partial derivative 9:  $X_{0}\leftarrow$ initial value                      ▷ setting the starting point 10:**end procedure** 11:**procedure**Finding optimal solution 12:  t← 0 13:  **while**
$i\neq N_{r}$**do**          ▷ Stop if the number of iterations exceeds the threshold 14:   **if**
$∥D(X)∥\leq \theta_{d}$**then**
break      ▷ Stop if the norm of gradient is sufficiently small 15:   **end if** 16:   $A_{T}\leftarrow$ tight constraints 17:   $A_{L}\leftarrow$ loose constraints 18:   **if**
$A_{T}\neq empty$
**then** 19:     $\mathrm{Pr}\leftarrow I-A_{T}^{\prime }{\left[A_{T}A_{T}^{\prime }\right]}^{-1}A_{T}$            ▷ Calculate projection matrix 20: 21:     $D^{\prime }\left(X_{i}\right)\leftarrow \mathrm{Pr}D\left(X_{i}\right)$ 22: 23:   **end if** 24:   **if**
$A_{L}\neq empty$
**then** 25:     $H\leftarrow -G\left(X\right)./A_{L}$ 26: 27:     λ←min（positive minimum element in H,1) 28:   **end if** 29:   $X_{t+1}\leftarrow X_{t}+\lambda D^{\prime }\left(X\right)$ 30:  **end while** 31:  **return**
final X                           ▷ optimal solution 32:**end procedure**


Here, it is noteworthy that the duty-cycle should be obligated not only in the uplink but also in the downlink. In other words, it takes a considerable time to properly adjust the data rate of all devices, especially in the case where a huge number of devices are deployed. Therefore, how long the network will take to update the entire SF should be discussed. Let $\alpha_{n}$ denote the ratio of devices that must update their SF, and then the gateway has to transmit $\alpha_{n}N$ packets to deliver the changed SF information through the downlink. In general, the downlink is available in one out of two reception windows, just after the uplink transmission. Therefore, the required time for the SF update is related to not only the duty-cycle in the downlink, but also the arrival rate of the devices.

We assume that all devices have the same arrival rate, λ, accordingly the *N* packets arrive at the gateway within 1/λ sec on average. For the sake of simplicity, the collisions are ignored. If the transmission of a gateway is not limited by the duty-cycle, the update can be completed within 1/λ sec. However, some devices cannot receive the update information due to the duty-cycle, consequently an SF update for those devices is conducted for next 1/λ sec. Meanwhile, $\alpha_{n}N$ packets can be transmitted every $\left(1/d-1\right)\alpha_{n}NT_{dl}\;sec$ from the gateway, where *d* is the duty-cycle of the gateway and $T_{dl}$ is the ToA of the downlink packet. As a result, we can roughly retrieve a lower bound of the time required for SF update using the following equation:

(25) $$m\left(1/d-1\right)\alpha_{n}NT_{dl}=k/\lambda ,$$

where *m* and *k* are arbitrary variables (m,k>0). By finding the minimum value of *m* and *k* satisfying the above equation, the minimum time for SF update can be calculated. We roughly explain the concept here, but it is sufficient to give an insight into the time required to complete the proposed method. The more detailed analysis and efficient solution for the SF update via downlink should be investigated in future works.

## 5. Numerical Results

### 5.1. Evaluation Environment

This section presents the numerical results of the proposed method. We calculated the total throughput with respect to the total number of devices ranging from 0 to 10,000. We assumed that the gateway knows the smallest available SF of each device by measuring the SNRs. Originally, ADR in the LoRaWAN was designed for static devices, so SNR variation is assumed to be small. Meanwhile, the number of devices using the large SFs should be relatively small in the total throughput optimization. Therefore, we supposed that just three kinds of SFs, i.e., SF7, SF8, and SF9 are used, for a clear explanation. It is reasonable because the devices far away from a gateway are likely to communicate with other nearby gateways. The channel occupation time per device is limited to the duty-cycle because LoRa works at the unlicensed band [17]. In EU863-870, the duty-cycle is 1%, accordingly we roughly decide the transmission probability of all the devices to be 0.01.

We denote $\alpha_{i}^{+}$ as the ratio of devices with the smallest available SF *i*. The motivation of our study is to optimize the throughput in environments where a large number of devices can use the same SF, thus the biased deployment must be considered. We set ($\alpha_{7}^{+}$, $\alpha_{8}^{+}$, $\alpha_{9}^{+}$) to (0.7, 0.2, 0.1). This can be understood that maximally 70% of the devices are allowed to use SF7, and 90% of the devices are allowed to use SF8 and so on. In the LoRaWAN, the maximum packet length can also be adjusted according to the used SF, but we assumed that all the devices transmit the same length of packet. For the comparison, we considered another two methods, uniform approach and naive approach. Uniform approach means that SFs are evenly assigned to the devices, i.e., setting ($\alpha_{7}$, $\alpha_{8}$, $\alpha_{9}$) to (0.33, 0.33, 0.33). Intuitively, this scheme adequately distributes the traffic load to all SFs. Meanwhile, the naive approach describes a method in which the devices directly use the smallest available SFs, i.e., (0.7, 0.2, 0.1). This method is intended to achieve high throughput by allowing all the devices to use the highest available data rate. It is also very similar to the behavior described in the current standard. In contrast to the comparisons using the fixed ratio, the proposed method dynamically derives ($\alpha_{7}$, $\alpha_{8}$, $\alpha_{9}$) according to the given network environments to maximize the throughput.

The optimization algorithm should be appropriately tuned according to the characteristic of the problem. Here, we focus on two design issues, scaling and local maxima problem. The gradient descent-based algorithms iteratively find the optimal solution by observing the current gradients until the observed gradients are almost zero. In this situation, scaling is very important. In detail, the value indicating the throughput is less than 10, but the number of devices ranges from 0 to 10,000. Therefore, the gradients are almost zero, regardless of the solution, accordingly the iterative process fails to find an optimal solution due to the early stopping. To deal with this problem, we adopt learning rate η, and the throughput multiplied by η is used instead of the original throughput shown in (12). In addition, we employed η as one tenth of the number of devices. This is due to the fact that the larger the number of devices, the longer it takes to find the solution due to the enlarged searching space. Therefore, the learning rate should increase in order to derive the optimal within the allowed time. Meanwhile, gradient descent-based algorithms inevitably face the local maxima problem. To find a global maximum, we simply use multiple initial points, and select the best solution among the multiple sub-optimal solutions independently retrieved from the initial points. In the evaluation, we use eight different initial points. For the summary, The used parameters are presented in Table 3.

### 5.2. Performance Evaluation

**Figure 5:**
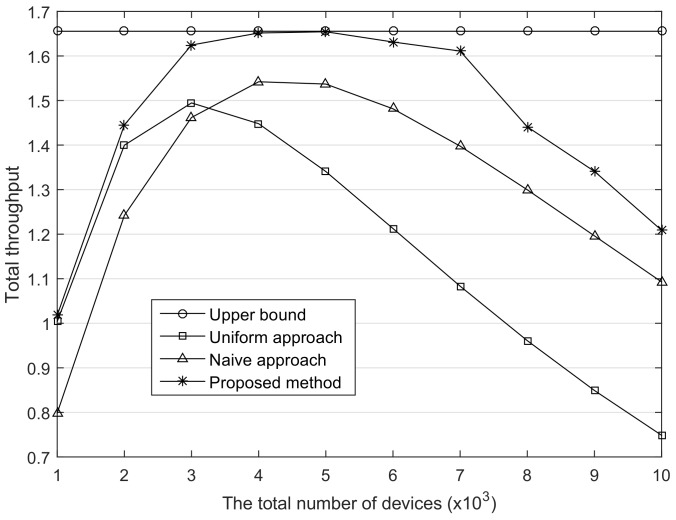
The comparison of the total throughput. The number of channel, $N_{c}$ is 3.

Figure 5 shows the total throughput according to the number of devices, assuming that the number of channels is 3. As shown in the figure, until the throughput reaches the peaks, they gradually increase. In addition, when the number of devices is small, the uniform approach is better than the naive approach owing to the less contention. However, as the number of devices increases, the naive approach is better than the uniform approach. This is because the smaller the SF used, the lower the rate of increase in the number of devices to the throughput reduction rate. Therefore, in the case that enormous devices are deployed at the network, it is recommended that a majority of devices uses as small of SFs as possible. Meanwhile, the proposed method provides higher performance than the others in all cases. Especially when the number of nodes is between 4000 and 6000, which leads to saturation throughput in the proposed method; the throughput is close to the theoretical upper bound. This result implies that the contention problem caused by the adjusted data rate must be appropriately handled. In addition, the ADR policy should be dynamically changed according to the network state.

**Figure 6:**
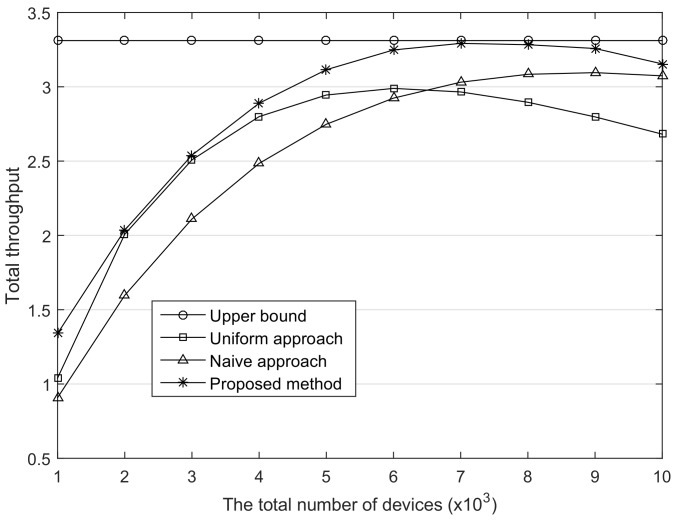
The comparison of the total throughput. The number of channel, $N_{c}$ is 6.

As the number of available channels increases, the networks can accommodate more devices owing to the less contention resulting from the channel diversity. Figure 6 shows the throughput in the same environment as the previous evaluation except for the number of channels; it changed from 3 to 6. Since the network capacity is proportional to the number of channels, the upper bound of throughput becomes double as compared with the previous one. It is noteworthy that the performance differences between the proposed method and the comparisons are more obvious when the number of devices is large. This means that the proposed method enhances the scalability in the LoRaWAN that considers a city scale area as a service coverage [18].

**Figure 7:**
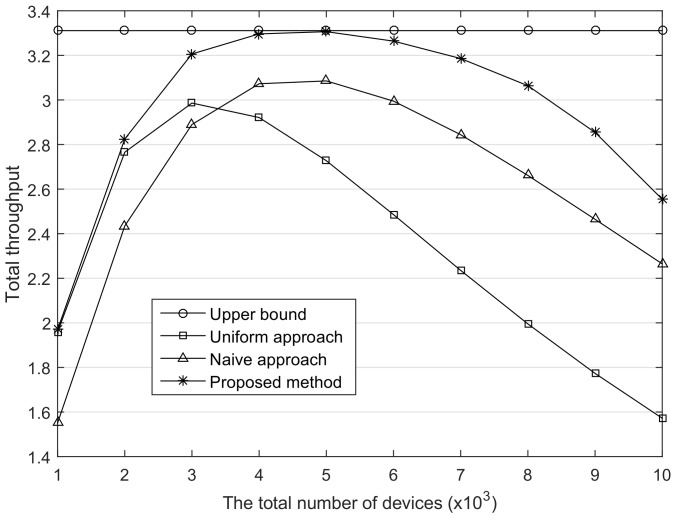
The comparison of the total throughput. The packet length is 100 byte.

In the LoRaWAN, the maximum MAC payload length is allowed from 59 to 230 bytes according to the used SF, and the smaller the factor is, the greater the payload length is allowed. Basically, the LoRaWAN technology is suitable for the exchange of small size data, but it may increase according to applications [19]. The ToA is proportional to the packet length, thus the traffic load on the network increases to exchange the large size packet, even with the same transmission probability. We changed the packet length from 50 bytes to 100 bytes and calculated the throughput as shown in Figure 7. Due to the growth of traffic load, the number of devices inducing the peak throughput is diminished. This reveals that the packet length can be a consideration in the throughput optimization. One suggestion is that it is a good strategy that reduces the packet length leading to less contention. In addition, based on the collaborative operation with those strategies, our method is able to flexibly adjust the data rate for the optimal throughput in the given network state.

**Figure 8:**
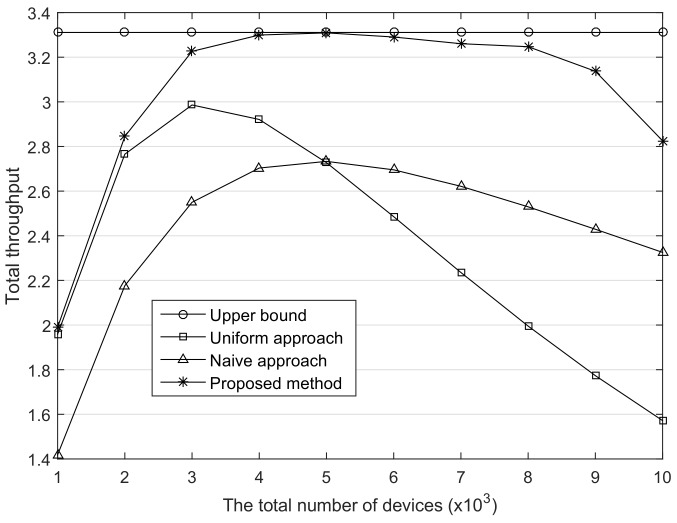
The comparison of the total throughput. The upper bounds of ratio of smallest $SF\left\{0.8,0.1,0.1\right\}$.

This paper assumes that only one gateway is dedicated to all devices. However, in terms of an empirical system, multiple gateways exist in the LoRaWAN and each device communicates with one of them. Therefore, the devices are more likely to join to the nearby gateway, and this may cause the more biased distribution of SF usage. To consider this environment, we set ($\alpha_{7}^{+}$, $\alpha_{8}^{+}$, $\alpha_{9}^{+}$) to (0.8, 0.1, 0.1) and evaluated our method. As shown in Figure 8, the throughput gaps between the proposed method and the others are greater as compared with the previous results. The average difference of the throughput between the proposed method and the naive approach is 0.37 in the previous environment, whereas it is 0.62 in this environment. Moreover, most of the presented throughput is close to the upper bound. This result indicates that in the case where multiple gateways are evenly deployed in the network, the advantage of the proposed method will be further emphasized.

## 6. Conclusions

The ADR provides a chance to optimize the total throughput in the LoRaWAN. Nevertheless, if most devices use the same data rate without consideration of the contention problem, the throughput may be reduced. This paper proposes contention-aware ADR to get an optimal throughput, and we find the optimal set via the gradient projection method. In particular, when a large number of devices have similar link quality, namely in the case of biased usage of the spreading factors, the proposed method can achieve considerably higher throughput than the current system owing to the load balancing effect.

The contributions provided in this study are the first step for the optimization of the LoRaWAN. In this paper, we focus on the numerical result achieved by the ADR. Therefore, practical issues should be considered in the next step, such as duty-cycle of downlink and consideration of all kinds of SF in the evaluation. Meanwhile, the main goal of this paper is throughput optimization. Therefore, the data rate is adjusted in the direction of increasing the number of devices using small spreading factors. Although this policy increases overall throughput, the transmission success ratio of the devices decreases. Accordingly, for applications in which reliability is more important than the throughput, our approach has to be supplemented. For future works, we will develop an optimization technique that takes into account the success ratio at the same time. In other words, our solution will be extended to the multi-objective optimization problem.

## Figures and Tables

**Table 1:** Data rate according to the SFs.

SF	$R_{b}$ (bps)	$R_{b}$ (bps) FEC with 4/5	$T_{s}$ (ms)
SF7	6835.94	5468	1.024
SF8	3906.25	3125	2.048
SF9	2197.27	1757	4.096
SF10	1220.70	976	8.192
SF11	671.39	537	16.384
SF12	366.21	292	32.768

**Table 2:** An example of how to set the upper bound of the number of devices which use a specific SF.

(a) Investigation of an available smallest SF in each device				
Device number	1	2	3	4
The available smallest SF	7	7	8	9
(b) The maximum number of devices permitted to use the SF				
SF	7	8		
The list of devices possible to use the SF	{ 1,2 }	{ 1,2,3 }	{ 1,2,3,4 }	
The upper bound(counting the components in each list)	2	3	4	

**Table 3:** Parameter setting.

Parameter	Value
Number of devices	0 to 10,000
Device type	Static
Available SF	SF7, SF8, SF9
Packet size	50, 100 byte
Number of channels	3, 6
Transmission probability	0.01
Number of initial points in the gradient projection method	8
Gradient threshold($\theta_{d}$)	$10^{-4}$
Maximum iteration number	$10^{6}$
